# Fibroblast diversity and plasticity in the tumor microenvironment: roles in immunity and relevant therapies

**DOI:** 10.1186/s12964-023-01204-2

**Published:** 2023-09-18

**Authors:** Yashi Xu, Wei Li, Shitong Lin, Binghan Liu, Peng Wu, Li Li

**Affiliations:** 1 Department of Obstetrics and Gynecology, Union Hospital, Tongji Medical College, Huazhong University of Science and Technology, Wuhan, Hubei China; 2 Cancer Biology Research Center (Key Laboratory of the Ministry of Education), Tongji Hospital, Tongji Medical College, Huazhong University of Science and Technology, Wuhan, Hubei China; 3 National Clinical Research Center for Gynecology and Obstetrics, Tongji Hospital, Tongji Medical College, Huazhong University of Science and Technology, Wuhan, China; 4 Department of Gynecologic Oncology, Tongji Hospital, Tongji Medical College, Huazhong University of Science and Technology, Wuhan, Hubei China

**Keywords:** Cancer-associated fibroblasts, Heterogeneity, Plasticity, Targeted therapies, Immunotherapies

## Abstract

Cancer-associated fibroblasts (CAFs), enriched in the tumor stroma, have received increasing attention because of their multifaceted effects on tumorigenesis, development, metastasis, and treatment resistance in malignancies. CAFs contributed to suppressive microenvironment via different mechanisms, while CAFs also exerted some antitumor effects. Therefore, CAFs have been considered promising therapeutic targets for their remarkable roles in malignant tumors. However, patients with malignancies failed to benefit from current CAFs-targeted drugs in many clinical trials, which suggests that further in-depth investigation into CAFs is necessary. Here, we summarize and outline the heterogeneity and plasticity of CAFs mainly by exploring their origin and activation, highlighting the regulation of CAFs in the tumor microenvironment during tumor evolution, as well as the critical roles performed by CAFs in tumor immunity. In addition, we summarize the current immunotherapies targeting CAFs, and conclude with a brief overview of some prospects for the future of CAFs research in the end.

Video Abstract

**Supplementary Information:**

The online version contains supplementary material available at 10.1186/s12964-023-01204-2.

## Introduction

Initially classified as cells that reside in connective tissue and generate collagen, fibroblasts are now characterized as interstitial cells of a mesenchymal lineage rather than as epithelial, endothelial, or immune cells [1]. Fibroblasts are functionally diverse, and quiescent fibroblasts aid in tissue structure maintenance, becoming activated when exposed to tissue damage and carcinogenesis [2–4]. Cancer associated fibroblasts (CAFs) are defined as those fibroblasts located within or adjacent to tumors.

CAFs constitute a major component of the stroma and play a pivotal role in tumor progression, therapeutic resistance, and immune evasion through the secretion of effective molecules and remodeling of the extracellular matrix (ECM) [5]. CAFs have long been regarded as tumor-promoting components, leading to the development of treatment strategies targeting CAFs. However, recent investigations have reported that targeting CAFs alone does not inhibit tumor growth and may even exacerbate cancer progression, resulting in poor clinical outcomes [6]. Advancements in novel co-culture models and single-cell RNA sequencing (scRNA-seq) technology have facilitated a deeper understanding of CAFs as highly heterogeneous mesenchymal lineage cells with different putative functions in distinct cancers [7]. Notably, emerging evidence suggests that CAF subtypes and proportions within tumors differ considerably in different stages of cancer, thereby influencing patient prognosis [4]. Furthermore, studies have demonstrated that treatment with JAK inhibitors can reprogram CAF subtypes in vivo, causing a shift from inflammatory CAFs (iCAFs) to myofibroblastic CAFs (myCAFs) [8]. Given that CAF transformation inevitably affects infiltrating immune cells, it also greatly impacts the tumor microenvironment (TME) and tumor immunity outcome. Therefore, further in-depth study of CAF subtype regulation during tumor progression is essential for designing effective combinatorial therapeutic approaches.

Here, we discuss and outline the heterogeneity and plasticity of CAFs, focusing on their establishment and activation, as well as their modulation within the TME during tumor progression and their crucial role in tumor immunity. We also provide an overview of currently used immunotherapies for the treatment of CAFs and conclude with a summary of potential directions for future CAF research.

## Origins and activation mechanisms of CAFs

**Fig. 1:**
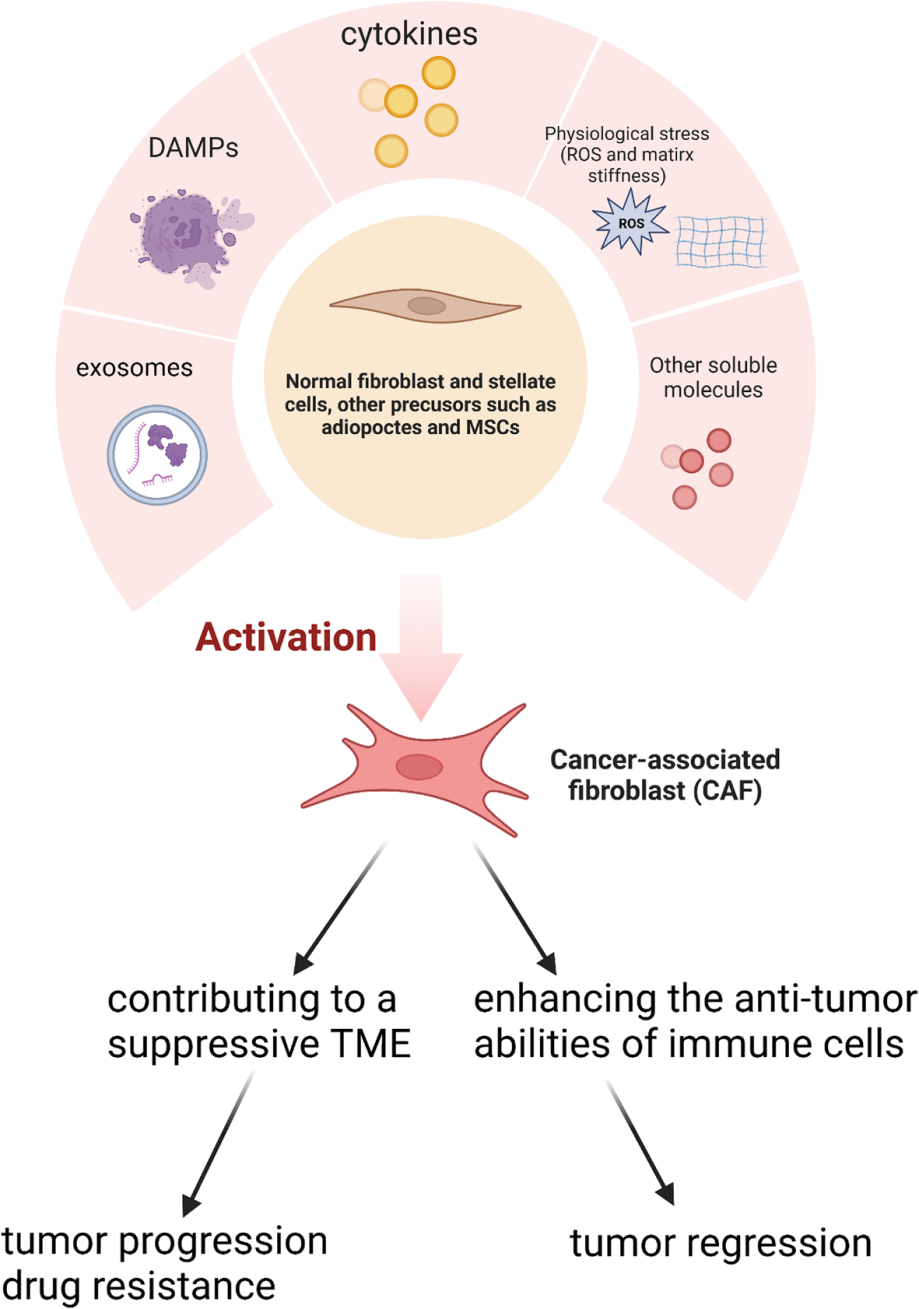
Origins, Activation of CAFs and General functions. Schematic description of various mechanisms involved in activation and general functions of CAFs. Potential cellular origins of CAFs include quiescent fibroblasts and stellate cells, bone marrow-derived mesenchymal stem cells (MSCs), adipocytes and other cell types. ECM, extracellular matrix; ROS, reactive oxygen species; DAMPs, damage associated molecular patterns

Dormant fibroblasts in tissue are activated during neoplasia, while progenitor cells are recruited from several sources (Fig. 1)and proliferate by activating different signaling pathways [9]. The arrangement of cells along the differentiation trajectory using pseudo-temporal approaches further illustrates stromal differentiation [4, 10, 11].

Tissue-resident fibroblasts are one of the main sources of CAFs [2]. Tissue-resident fibroblasts are recruited and stimulated by different modulators in various malignancies, consisting of transforming growth factor (TGF)-β [12], stromal cell-derived factor-1 (SDF-1) [13], hepatocyte growth factor (HGF) [14], platelet-derived growth factor (PDGF) [15], and reactive oxygen species (ROS) [16]. Stellate cells are an additional source of CAFs in some tumors [17, 18]. Vitamin A shortage contributes to the activation of pancreatic stellate cells (PSCs) [19], whereas stimulation of insulin-like growth factor 1 (IGF-1) signaling is essential for the activation of hepatic stellate cells (HSCs) [20].

Mesenchymal stem cells (MSCs) are suggested to serve as CAF precursors [4, 21]. Notably, the transformation of MSCs into CAFs can be induced by the secretion of TGF-β1 from cancer and stromal cells [22]. Further studies have also highlighted the importance of the TGF-β signaling pathway in the transition of MSCs to CAFs [23, 24], while in vitro experiments have demonstrated that MSCs can acquire CAF-like characteristics upon persistent stimulation with tumor necrosis factor (TNF)-α and interleukin (IL)-1β [25]. Intriguingly, macrophages can assist MSCs in developing CAF-like features [26]. Furthermore, the differentiation of bone marrow MSCs into CAFs, triggered by cancer cells, is primarily dependent on the Notch and Akt signaling pathways [27], implying that some intrinsic signaling pathways may play a role in MSC transformation.

Adipocytes are also considered as a type of CAF precursor [28]. For instance, human adipose tissue-derived stem cells (HASCs), stimulated by TGF-β1, can transdifferentiate into CAFs with a fibroblastic characteristic (α-smooth muscle actin (SMA)( +), tenascin-C( +)) [29]. Other cell types, such as epithelial cells [30], pericytes [31], monocytes [32], endothelial cells [33], mesothelial cells [34], hematopoietic stem cells [35], circulating bone marrow cells [36], and smooth muscle cells [37], are also reported as potential CAF precursors. Furthermore, CAF activation can be triggered by damage-associated molecular patterns(DAMPs)released from damaged tissues or dying cancer cells [38]. Lysophosphatidic acid, released by hepatocellular carcinoma (HCC) cells, can promote the differentiation of peritumoral tissue fibroblasts into a CAF-like myofibroblastic phenotype [39]. Tumor-derived exosomes that contain different molecules, such as miR-192/215 family microRNAs (miRNAs), can also promote CAF-like differentiation in head and neck squamous cell carcinoma [40]. Finally, activation of CAFs can also be impacted by environmental stressors, such as ROS [41], and matrix stiffness [42].

CAFs represent a highly heterogeneous cell population, with extensive evidence supporting the existence of multiple cellular progenitors and diverse activation routes. Activated CAFs exhibit dual effects on tumor development, both promoting and inhibiting tumor growth. However, further research is required to fully map the landscape of CAF subpopulations across human malignancies.

## Heterogeneity of CAFs in different cancers and at different stages

The molecular and functional diversity of CAFs arises from diverse sources and activation mechanisms. The advancement of scRNA-seq technology has revolutionized our understanding of CAFs, revealing their complex heterogeneity across different tumors and in distinct phases within the same malignancy (Table 1).

**Table 1:** Phenotypic and functional heterogeneity of cancer-associated fibroblasts in different cancers and at different stages

Cancer types	Subtypes	Characteristic markers	Functions	supplementary	Ref
PDAC	iCAFs	HAS1, HAS2, AGTR1, IL-6, IL-11, CXCL1, CXCL2, Lif, PDGFRα	immunosuppressive		[17]
	myCAFs	αSMA, CTGF, TNC, TAGLN	ECM producing		
PDAC	rCAFs	Meflin	cancer-restraining abilities		[43]
PDAC	CD105^pos^	CD105, other like FSP1, GPR77	needs further characterization		[44]
	CD105^neg^	FSP1, GPR77	restrict tumor growth		
PDAC	apCAFs	MHC class II and CD74, H2-Aa, H2-Ab1	antigen-present, immune-modulatory capacity		[45]
PDAC	iCAFs	FAP, ACAT2, COL3A1, CXCL12	immunosuppressive	only in PDAC	[46]
	myCAFs	FAP	not defined	exist in all stages	
PDAC	CAF-A	Periostin	tumor proliferation, invasiveness, metastasis		[47]
	CAF-B	Myosin-11	not defined		
	CAF-C	PDPN	an indicator of immunogenic tumors, immune promotion		
	CAF-D	not mentioned	poor prognosis		
BC	CD10 + GPR77 + CAF	CD10 and GPR77	chemoresistance		[48]
BC, HGSOC	CAF-S1	CD29^Med^ FAP^Hi^ FSP1^Med^ αSMA^Hi^ PDGFRβ^Med−Hi^ CAV1^Low^	tumor proliferation, migration, lymph-nodes metastasis, immune suppression and EMT initiation		[49, 50]
	CAF-S2	Low expression of most detected markers	not defined		
	CAF-S3	FAP^Neg^ αSMA^Neg^ CD29^Med^ FSP1^Med−Hi^ PDGFRβ^Med^ CAV1^Low^	not defined		
	CAF-S4	CD29^Hi^ FAP^Neg^ FSP1^Low−Med^ αSMA^Hi^ PDGFRβ^Low−Med^ CAV1^Low^	tumor invasion, migration, lymph-nodes metastasis		
BC	Vascular CAF	αSMA and PDGFRβ	angiogenesis		[51]
	Matrix CAF	Fibulin 1 and PDGFRα	immune regulation		
	Cycling CAF	PDGFRβ	not defined		
	developmental CAF	SCRG1, SOX9	angiogenesis		
BC	PDPN + CAF	6 subtypes: immune reg E (CXCL12), immune reg L (SAA3), ECM (fibrillin 1), wound healing (αSMA), inflammatory A (CXCL1) and inflammatory B (IL-6)	changing with cancer development	The composition of CAF subtypes varies with disease progression	[4]
	S1004A + CAF	2 subtypes: protein folding (HSPD1) and antigen presenting (SPP1)			
CRC	CAF-A	FAP, MMP2 and DCN	ECM remodeling		[52]
	CAF-B	αSMA, TAGLN and PDGFA	not defined		
GC	STF1	FAP, ACTA2, and TAGLN	not defined	stage-dependent increase	[53]
	STF2	CSPG4, INHBA	not defined		
	STF3	FAP, ACTA2, and TAGLN, INHBA	poor prognosis		
PTC	iCAFs	CFD, PLA2G2A, CCDC80	recruiting and crosstalking with diverse immune cells	stage-dependent increase	[54]
	myCAFs	αSMA, TAGLN, MYLK, MYL9	exert mechanical and chemical influence on tumor progression		

*PDAC* Pancreatic ductal adenocarcinoma, *BC* breast cancer, *HGSOC* high-grade serous ovarian cancer, *CRC* colorectal cancer, *GC* gastric cancer, *PTC* Papillary Thyroid Carcinoma, *HAS1* Hyaluronan Synthase 1, *AGTR1* angiotensin II receptor type 1, *IL* interleukin, *CXCL* chemokine (C-X-C motif) ligand (CXCL), *PDGFR* Platelet Derived Growth Factor Receptor, *αSMA/ ACTA2* Actin Alpha 2, Smooth Muscle, *CTGF* Connective tissue growth factor, *TNC* Tenascin C, *TAGLN* transgelin, *CD* cluster of differentiation, *FSP1* ferroptosis suppressor protein 1, *GPR77* G protein-coupled receptor 77, *MHC* major histocompatibility complex, *H2-Aa/ H2-Ab1* histocompatibility 2, class II antigen A, alpha/beta1, *COL3A1* Collagen Type III Alpha 1 Chain, *FAP* fibroblast activation protein, *Myosin-11* Myosin Heavy Chain 11, *PDPN* Podoplanin, *CAV* caveolin, *SCRG1* Scrapie responsive gene one, *SOX9* SRY-Box Transcription Factor 9, *SAA3* Serum amyloid A-3, *HSPD1* Heat Shock Protein Family D (Hsp60) Member 1, *SPP1* Secreted Phosphoprotein 1, *CSPG4* Chondroitin Sulfate Proteoglycan 4, *INHBA* Inhibin Subunit Beta A, *MMP2* Matrix Metallopeptidase 2, *DCN* Decorin

Ӧhlund et al. first identified two opposite subtypes of CAFs—myCAFs and iCAFs, which might serve to tumor progression in pancreatic cancer. MyCAFs are in the vicinity of cancer cells and characterize by high α-SMA expression, while iCAFs are away from cancer cells and secrete inflammatory factors [17]. In subsequent research, Mizutani and colleagues [43] discovered a subset of CAFs (termed cancer-restraining CAFs (rCAFs)) in pancreatic ductal adenocarcinoma (PDAC) expressing meflin and showing anticancer effects in both animal models and human cells. CD105-expressing fibroblasts are also recognized as highly tumor suppressive [44] in PDAC, while a newly identified CAF subpopulation, termed antigen-presenting CAFs (apCAFs), induces T cell anergy or T regulatory cell differentiation in PDAC and breast cancer (BC) [34, 45]. Interestingly, primary human lung apCAFs derived from ATII rather than cancer or mesothelial cells [44, 55] exhibit certain anti-tumor effects by directly activating T cell receptors (TCRs) in tumor-infiltrating CD4 + T cells and shielding them from apoptosis. Notably, in addition to the phenotypic and functional variations in CAFs, the proportion of different CAF subtypes also fluctuates with tumor progression. The emergence of iCAFs has been implicated in immune evasion during the multistep progression from intraductal papillary mucinous neoplasms to PDAC [46] and in mouse models [56]. Studies have also assessed the intertumoral and intratumoral heterogeneity of human PDAC-derived CAFs [47], with the CAF-C subtype identified as a potential indicator of immunogenic tumors compared to other subtypes in human PDAC. CAFs in different metastatic niches also present different degrees of heterogeneity in PDAC [57].

Studies have also identified different CAF subpopulations in human BC [58]. CAF subsets, expressing CD10/Gpr77 and Hedgehog target genes respectively, promoted the characteristics of cancer stem cells [48, 59] and contributed to chemoresistance in BC. These CAF populations also exhibit stage-specific heterogeneity in BC. Four CAF subsets (CAF-S1–S4) have been identified in BC and high-grade serous ovarian cancer (HGSOC) [49, 50]. The CAF-S1 and CAF-S4 subsets appear at a high level in aggressive BC (HER2 and triple negative) and metastatic lymph nodes. In contrast, the CAF-S2 subset is enriched in the luminal BC subtype, while the CAF-S3 subset is more prevalent in healthy tissues. These CAF subpopulations not only interact dynamically in BC tissue [51], but their composition also varies dynamically during BC progression [4]. Multiple CAF subpopulations with distinct phenotypes have also been detected in colorectal cancer (CRC) [52]. Recent studies have also validated shifts in the proportional representation of CAF subtypes during disease progression in papillary thyroid carcinoma (PTC) and gastric cancer (GC) [53, 54].

In conclusion, CAFs exhibit heterogeneity across different tumors and even within different stages of the same carcinoma. It is postulated that CAF subtypes represent diverse intermediate states rather than fully differentiated end states. However, additional studies are needed to further dissect the heterogeneity of CAFs.

## Modulation of CAFs during tumor progression

**Fig. 2:**
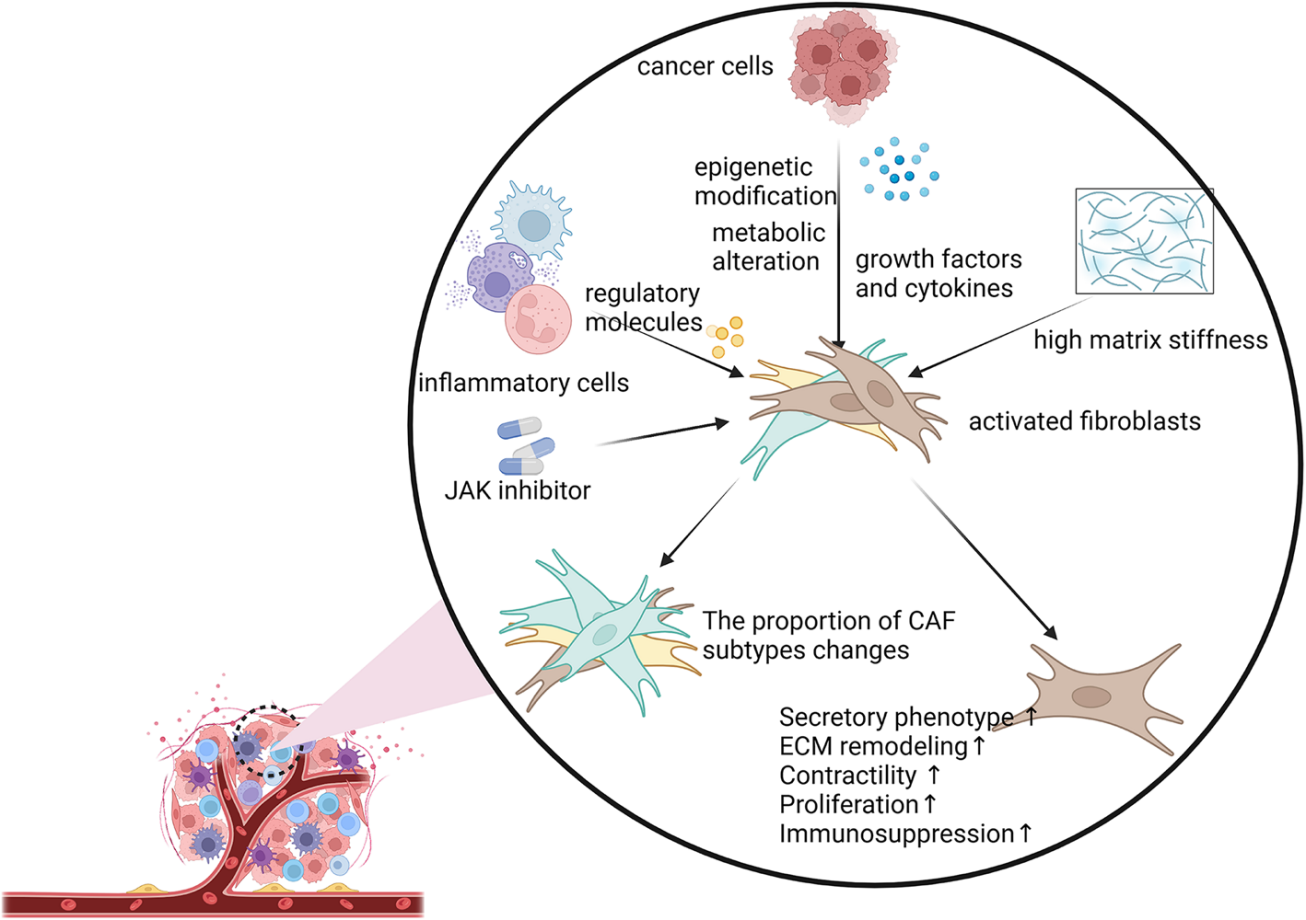
Modulation of CAFs. The variables and possible potential mechanisms that affect the alteration of CAFs in the microenvironment are briefly summarized. The morphology and function of CAFs vary as the tumor progression due to numerous variables in the tumor microenvironment. Cancer cells can modulate CAFs by releasing chemical compounds, altering metabolisms and epigenetic modification. Similarly, regulatory molecules released by inflammatory cells also have an impact on CAFs. Besides, the extracellular matrix exerts a great regulatory effect on CAFs

CAFs display considerable plasticity and undergo dynamic transformations during tumor progression [17, 45, 52–54, 60]. This may stem from the diverse origins of CAF precursor cells in different malignancies. However, it is also plausible that CAFs are regulated by various elements within the TME, and thus show considerable plasticity during tumor development. Here, we briefly discuss the variables and possible mechanisms that may regulate the mutual transformation of CAF subtypes in the TME (Fig. 2), providing potential insights into the development of targeted CAF therapy aimed at limiting tumor growth, as dynamic changes in CAFs are directly related to tumor development.

### Cancer cell-mediated fibroblast modulation

#### Modulation of fibroblasts by epigenetic modification

Alterations in CAF phenotypes and functions may result from the externalization of hallmark CAF genes. Previous integrative mutational analysis identified 14 and 413 genes related to the degree of myCAF and iCAF differentiation, respectively [61]. CAF regulation is also closely connected to epigenetic modifications within CAF characteristic genes, including DNA methylation, histone modification, nucleosome remodeling, and RNA-mediated targeting [62].

DNA methylation patterns have been reported between prostatic normal tissue-associated fibroblasts and CAFs, revealing hypermethylation in the promoters of 18 tumor-promoting, 11 suppressing, and two metastasis regulatory genes in CAFs in prostate cancer [63]. PDAC with organ-specific metastatic potential exhibits distinct capabilities in terms of methylating metabolism genes in CAFs, modulating CAF phenotypes, and generating different CAF heterogeneities within different metastatic niches [57]. Moreover, in BC and PDAC, mesenchymal phenotypes are influenced by genomic instability and mutation status of neoplastic cells [64, 65]. Notably, previous research has confirmed that CAFs revert to normal fibroblasts when histone methylation is reduced [66]. Thus, how genetic abnormalities in neoplastic cells shape CAF heterogeneity and plasticity will be an essential element in future CAF research.

Extracellular vesicles (EVs) and miRNA dysregulation generated from tumor cells are reported to play a role in CAF regulation [67]. For example, both early- and late-stage colorectal cancer cell-derived exosomes can activate quiescent normal fibroblasts (α-SMA( −), CAV( +), FAP( +), VIM( +)) into CAF-like fibroblasts (α-SMA( +), CAV( −), FAP( +)). However, pro-angiogenic and pro-proliferative protein synthesis is increased when fibroblasts are stimulated by early-stage cancer exosomes, whereas those stimulated by late-stage cancer exosomes infiltrate the ECM via activation of pro-invasive membrane protrusion regulators and matrix-remodeling proteins [68]. Previous miRNA profiling of EVs has implicated various miRNAs in the generation of chemokines such as chemokine (C-X-C motif) ligand(CXCL)1 and CXCL8 in fibroblasts, correlated with poorer survival in gastric cancer patients [69]. In addition, unlike mesenchymal colorectal cancer(CRC) EVs, epithelial CRC EVs, rich in miR-200, have been shown to inhibit TGF-β-driven myofibroblast differentiation [70]. These findings demonstrate that CAFs can be influenced by different active chemicals produced and released by tumor cells at different stages of tumor formation, leading to corresponding morphological and functional modifications that promote tumor growth. Future research should focus on understanding the mechanisms in greater detail.

#### Modulation of fibroblasts by transcription factors

Transcription factors (TFs) are a class of regulatory proteins that modulate the expression of target genes by binding to specific *cis*-regulatory elements, thereby influencing important biological functions. In triple negative breast cancer (TNBC), several TFs have been identified to control the differentiation states of stromal cells [71]. For instance, paired related homeobox 1 (PRRX1) orchestrates the functional transition of fibroblasts into a myofibroblastic phenotype via the TGF-β signaling pathway by remodeling a super-enhancer landscape [72]. Furthermore, in prostate cancer, complicated interactions between the ELF3 and YAP/TAZ restricts the transdifferentiation of iCAFs into myCAFs via the classical TGF-β1 pathway [73]. In addition, overexpression of the TCF21 in CAFs with high FAP, which is exclusive to low levels of FAP, can impair the ability of CAFs to promote tumor invasion, chemoresistance, and progression [74].

#### Modulation of fibroblasts by growth factors and cytokines

Growth factors and cytokines that modulate and activate stromal fibroblasts are present in large quantities in cancer cells. Among these factors, TGF-β is a well-studied and universally expressed cytokine that plays diverse roles in cancer progression, metastasis, and management [75], as well as the activation and regulation of CAFs. Öhlund et al. [17] demonstrated that iCAFs and myCAFs can be produced under various trans-, mono-, and co-culture settings, indicating that they are phenotypically reversible in response to various growth stimuli. Similarly, apCAFs can differentiate into myCAFs upon culture [45]. Biffi et al. [8] found that induction of IL-1 by tumor cells stimulates the Janus kinase (JAK)/signal transducer and activator of transcription (STAT) signaling pathway through up-regulation of leukemia inhibitory factor (LIF), leading to the formation of the iCAF phenotype, while tumor-secreted TGF-β inhibits IL-1-induced JAK/STAT signaling, resulting in the suppression of iCAF activation and enhancement of myCAF features. These findings suggest that IL-1 signaling regulates iCAF differentiation in cancers [8]. A novel subtype of CAFs, known as interferon-licensed fibroblasts (ilCAFs), which are characterized by their remarkable response to interferons, has also been discovered in BC [76]. Neutralizing TGF-β leads to the reduction of myCAFs and the expansion of ilCAFs within the stroma, presenting a potential therapeutic strategy to target CAF differentiation and harness their immunomodulatory effects [76]. Furthermore, the Hedgehog signaling pathway is reported to specifically activate fibroblasts, with myCAFs displaying significantly higher activation compared to iCAFs. Manipulating the Hedgehog pathway can decrease the abundance of myCAFs while increasing the presence of iCAFs, thus suppressing Hedgehog signaling in CAFs [77]. Additionally, LIF can stimulate fibroblasts to undergo internal epigenetic modifications, thus sustaining the pro-invasive characteristics of CAFs by activating the JAK1/STAT3 signaling pathway [78].

In conclusion, it is evident that cytokines within the TME influence phenotypic and functional diversity of CAFs by triggering multiple downstream signaling pathways. However, the specific cytokines involved and the precise mechanisms by which they regulate CAFs during tumor development and metastasis remain incompletely understood. Therefore, further investigations are necessary to elucidate the underlying mechanisms and expand our understanding.

#### Modulation of fibroblasts via metabolic adaptations

The establishment of CAFs is a crucial step in cancer initiation and progression. Tumor cells primarily regulate signaling pathways, like autocrine loops with CAF metabolic reprogramming, leading to the constitutive activation of tumor stroma and the differentiation of CAFs in numerous solid malignancies [16, 76]. Tumors frequently exhibit a metabolic shift towards aerobic glycolysis, while neighboring fibroblasts, upon activation by cancer cells, undergo differentiation into CAFs. Furthermore, under ROS exposure, fibroblasts acquire an increased migratory capacity through the accumulation of HIF1α and CXCL12 [77]. In addition, TGF-β1 or PDGF can trigger the metabolic conversion of CAFs from oxidative phosphorylation to aerobic glycolysis by down-regulating isocitrate dehydrogenase 3 [78]. While research on CAFs has advanced considerably in recent years, studies focusing on metabolic variations among the various subtypes of CAFs remain scarce. At various stages of tumor formation, cancer cells continuously regulate CAFs through multiple mechanisms, such as metabolic adjustment. Modified CAFs, in turn, contribute to tumor development, suggesting a novel mechanism for developing future therapeutic strategies targeting tumors.

### Inflammatory cell-mediated fibroblast modulation

Inflammatory cells play a vital role in regulating the TME, which is now acknowledged as a significant contributor to the neoplastic process by promoting cellular proliferation, survival, and migration [79]. CAFs and inflammatory cells coexist in spatial and temporal proximity throughout tumor development, indicating a subtle regulatory connection between them.

Tumor-associated macrophages (TAMs), a group of macrophages that infiltrate tumors, are categorized into two main subtypes: i.e., M1 with anti-tumor effects and M2 with pro-tumor effects [80]. Multiple studies have demonstrated that CAFs facilitate the recruitment of monocytes and their differentiation into the M2 subtype, leading to an immunosuppressive TME across various cancers [81–83]. Reciprocally, M2 macrophages further regulate CAF activation and progression [84, 85], while TAMs also participate in MSC transdifferentiation and activity [86]. Although studies on the impact of CAFs on TAMs have been reported, the impact of macrophages on CAFs has not yet been thoroughly examined and defined.

Recent studies have shifted focus towards the role of mast cells (MCs) in cancer, rather than solely focusing on their functions in allergy-related illnesses [87]. Intriguingly, MCs exert dual effects on tumor development, displaying both tumor-promoting [88] and tumor-inhibiting properties [89], depending on precise MC localization, cancer type, and tumor stage [90–92]. The coexistence of elevated levels of CAFs and MCs in tumor islets is closely correlated with cancer aggressiveness, with their interactions directly promoting tumor growth [93, 94]. Notably, MCs secrete tryptase and IL-13, which promote CAF proliferation [95], while CAFs can potentiate MC proliferation, migration, and inflammatory cytokine secretion, thus exhibiting pro-tumorigenic effects [96]. Additionally, MCs in neurofibroma enhance CAF activity by up-regulating CAF secretion and proliferation through the TGF-β signaling pathway, thereby boosting the tumor-promoting effects of CAFs [93].

Overall, the precise mechanisms underpinning the intricate interactions between CAFs and other cells remain largely unknown. In addition, there is still a substantial gap in our understanding of how immune cells modulate CAFs.

### ECM-mediated fibroblast modulation

In both normal and malignant tissues, the bidirectional communication between cells and the extracellular environment is crucial [97]. In three-dimensional (3D) cultures, increased matrix stiffness promotes the differentiation of fibroblasts into myofibroblasts as well as the synthesis of matrix proteins [98]. This effect is further potentiated by TGF-β, indicating a cooperative relationship between TGF-β and matrix stiffness to promote fibroblast differentiation [99]. Furthermore, Yes-Associated Protein (YAP) is necessary for CAFs to induce matrix stiffening, which further enhances YAP activation and establishes a feedforward self-reinforcing loop that helps sustain the CAF phenotype [100]. The up-regulation of α-SMA expression, stimulated by this positive feedback loop, enhances the contractility of myofibroblasts, resulting in increased traction on the fibrotic ECM, leading to further matrix remodeling and stiffening. This, in turn, promotes potential TGF-β activation, culminating in a vicious cycle [101]. Furthermore, the Dickkopf 3 (DKK3) protein, a heat shock factor 1 target gene, also plays a vital role in promoting aggressive behaviors in CAFs by amplifying YAP/TAZ activity via the canonical Wnt signaling pathway [102]. However, further research is necessary to determine the processes by which CAFs, after regulation by the ECM, can influence the TME and impact tumor growth, as well as possible therapeutic target molecules in this process.

The abovementioned findings indicate that the pattern and function of CAFs are subject to constant adjustment due to multiple variables within the TME and tumor load fluctuation. Various factors contribute to the heterogeneity and plasticity of CAFs. Therefore, further investigation of the roots and mechanisms of regulation is warranted.

## Roles of CAFs in immunity

CAFs play a pivotal role in orchestrating tumor-promoting environments through complex signaling interactions with cancer cells, matrix components, and infiltrating immune cells. On the one hand, the establishment of CAFs is accompanied by a decrease in cytotoxic T cells, a decline in killing capacity, and an increase in myeloid suppressor cells, leading to the immunosuppressive TME observed in many malignancies [46, 53, 54, 103]. In addition, the remodeling effects of CAFs on the ECM restrict the accessibility of tumor-killing cells and block the infiltration of therapeutic agents. On the other hand, CAFs also exert anti-tumor effects through various mechanisms [104–106]. Different CAF subsets exhibit distinct functions under specific circumstances, leading to different clinical outcomes in patients as well as tumor regression. This heterogeneity in CAF subtypes forms the basis for certain targeted CAF therapies.

### Tumor-promoting functions of CAFs

**Fig. 3:**
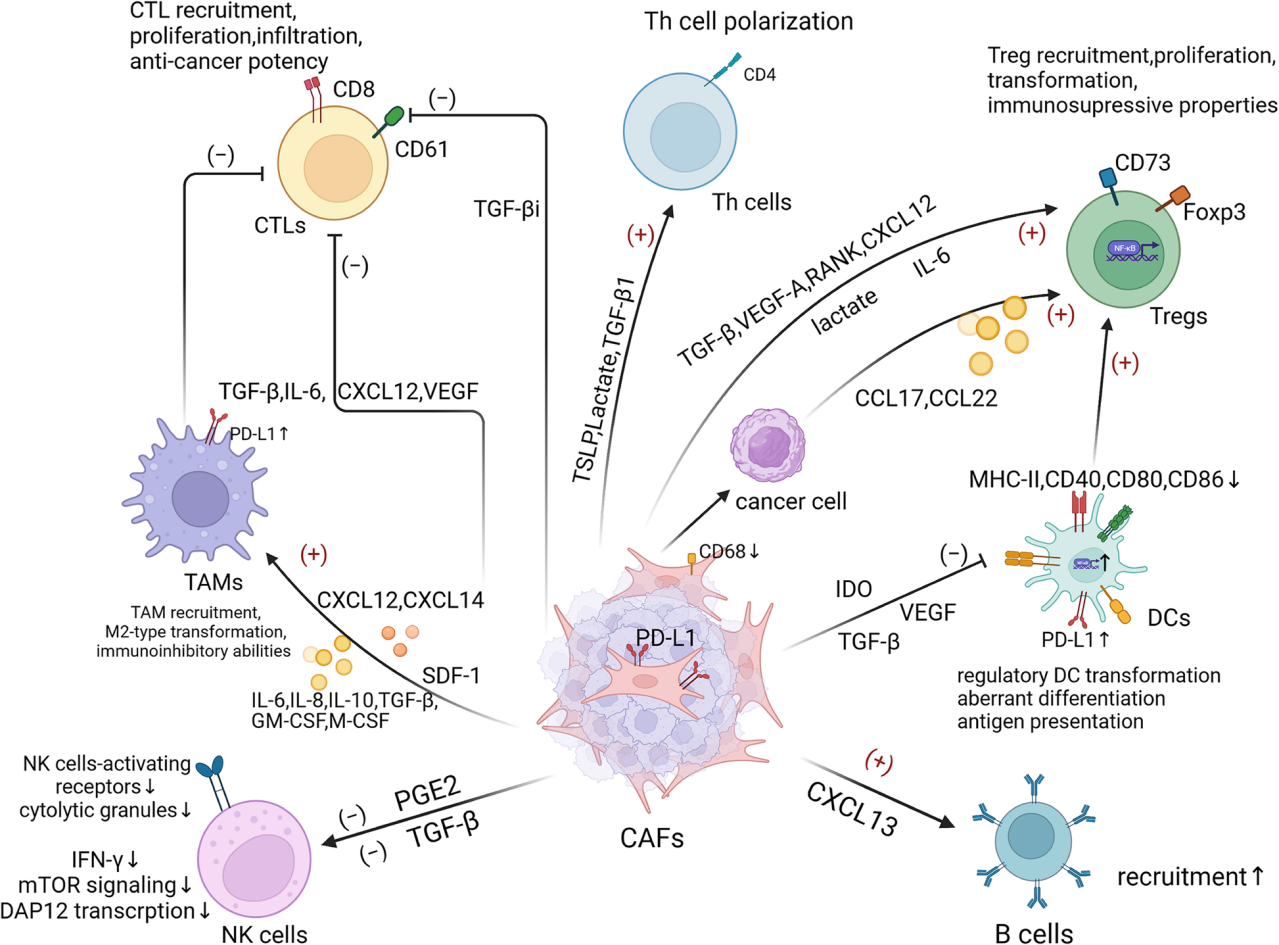
Regulation of CAFs on immune cells. CAFs can orchestrate an immunosuppressive TME by interacting with the immune cells in tumors. By secreting a variety of chemokines, cytokines and other effector molecules, such as TGF- β, IL-6, C-X-C chemokine ligand 12 (CXCL12), C–C-C chemokine ligand 2 (CCL2), SDF-1, vascular endothelial growth factor (VEGF), indoleamine 2,3-dioxygenase (IDO) and prostaglandin E2 (PGE2), CAFs regulate immune cells-mediated anti-tumor immunity in tumor microenvironment via triggering multiple pathways

Here, we discuss the mechanisms through which CAFs facilitate tumor growth by regulating immunocytes within the TME (Fig. 3).

#### Regulatory effects of CAFs on TAMs

As previously mentioned, TAMs can be categorized into the M1 and M2 subtypes [107]. M1-type macrophages primarily exhibit anti-tumor activity in the TME by mediating antibody-dependent cellular cytotoxicity and producing ROS and TNF [80], whereas M2-type macrophages exhibit tumor-promoting activity by assisting in tumor angiogenesis, immunosuppression, cancer cell invasion and metastasis, and ECM remodeling [108, 109]. TAMs are the most abundant inflammatory cells in CAF-populated regions, highlighting the close interactions between these two cell types [82, 110]. Multiple studies have shown that CAFs promote the recruitment of monocytes (macrophage precursors) [111–114] and induce immunosuppressive M2-like TAMs through the production of cytokines, including IL-6, IL-8, IL-10, TGF-β, GM-CSF, and M-CSF [111, 115–118], and chemokines, such as CXCL12 [82] and CXLC14 [119]. Recently, a new subtype of macrophage, STAB1 + TREM2^high^ lipid-associated macrophage (LAM), has been identified [120]. Monocytes recruited to the tumor site are transformed into LAM to support the immunosuppressive microenvironment via the CAF-driven CXCL12-CXCR4 axis in TNBC. Gordon et al. [121] observed increased expression of programmed cell death protein 1 (PD-1) on the cell surface of CAF-induced M2 macrophages, with subsequent studies indicating that TAMs with high PD-1 expression can suppress the innate and adaptive anticancer immune responses, including reducing their own phagocytic potency against tumor cells and preventing T-cell infiltration and proliferation [118].

#### Regulatory effects of CAFs on dendritic cells (DCs)

Tumor-infiltrating DCs are a potent and versatile population of specialized antigen-presenting cells that play a key role in activating and regulating the immune response in TME [122]. Several recent studies have shown that CAFs can drive immune evasion of tumor cells by affecting DC maturation, antigen presentation, and associated immune responses, although the mechanisms involved remain unclear. Notably, TGF-β secreted by CAFs can down-regulate the expression of MHC class II molecules and co-stimulatory molecules CD40, CD80, and CD86 on the surface of DCs. These immature cells promote the formation of regulatory T cells (Tregs), which inhibit effector T cell function [123]. Additionally, CAFs in hepatocellular carcinoma can promote the development of regulatory DCs, characterized by low expression of co-stimulatory molecules and high production of suppressive cytokines, thus promoting T cell exhaustion and Treg proliferation via IDO overexpression [124, 125]. In addition, CAF-generated VEGF contributes to aberrant differentiation of DCs and reduces antigen-presenting ability through NF-κB activation [126, 127], and promotes immunological tolerance by increasing PD-L1 expression on the DC surface [128]. Furthermore, under stimulation by tumor-derived TNF and IL-1, CAFs can produce thymic stromal lymphopoietin (TSLP), which promotes Th2 cell polarization through myeloid DC training [129].

#### Regulatory effects of CAFs on natural killer (NK) cells

As cytotoxic lymphocytes of the innate immune system, NK cells can recognize and kill tumor cells through killer ligands [130]. Various studies have demonstrated that CAFs can suppress NK cells through a range of mechanisms, such as NK receptor activation [131] and cytotoxic capability. For example, in melanoma cancer, CAF-secreted prostaglandin E2 (PGE2) can reduce the expression of activation receptors on the NK cell surface as well as the production of cytolytic granules [132]. In colon cancer, NK cells can promote the formation of CAF-induced inhibitory loops by promoting the secretion of PGE2 [133]. TGF-β, a widely studied cytokine, also plays a key role between CAFs and NK cells. Notably, CAF-secreted TGF-β can limit NK cell activation and cytotoxic potential and can down-regulate activation receptors on NK cell surfaces and reduce interferon-gamma (IFN-γ) production [134, 135] by inhibiting DNAX-activation protein 12 (DAP12) transcription [136] and mTOR signaling pathway activation [137]. In addition, CAF-induced macrophages can act synergistically with CAFs to inhibit NK cell function [111].

#### Regulatory effects of CAFs on T cells

T lymphocytes play a central role in the regulation of adaptive immune responses and consist of different subpopulations, including helper T (Th) cells, regulatory T cells (Tregs), and cytotoxic T lymphocytes (CTLs). Many studies have illustrated the role of CAFs in the regulation of T cell activity and function.

An effective immune response relies on the priming of tumor-specific CD8 + T cells and CD4 + Th1 cells. Recent research has uncovered a potential suppressive mechanism employed by CAFs in the TME, wherein they exhibit DC-like functions such as antigen collection, processing, and presentation, and up-regulation of immune checkpoint molecule expression [138]. Furthermore, increased TGF-β production and PD-L1 and PD-L2 expression in CAFs can contribute to the suppression of T cell proliferation [139].

Th cells, which are derived from naïve CD4 + T cells [140], mainly include Th1, Th2, and Th17 cells, which participate in cellular and humoral immunity [141]. Several studies have shown that CAFs contribute to Th cell recruitment [142, 143] and polarization [144, 145], although their precise effects remain unclear. CAFs can also facilitate Th17 cell differentiation by producing TGF-β1 during tumor progression [146].

Tregs are critical for tumor immunity, particularly through the expression of FOXP3, which has a significant inhibitory effect on the anti-tumor immune response [147]. Penetration of the spatially connected Foxp3 + Tregs and CAFs in the tumor stroma is strongly associated with poor prognosis [148]. CAF-derived TGF-β can promote the differentiation of naïve T cells into CD4 + CD25 + Tregs by inducing Foxp3 gene expression in T lymphocytes across different malignancies [145, 149]. In addition to influencing the differentiation of primitive T cells into Treg cells, CAFs can stimulate the migration of Tregs and significantly increase their frequency at the tumor site by secreting chemokines and regulatory molecules [49, 50, 150–152]. Moreover, down-regulating CD68 in CAFs enhances the release of CCL17 and CCL22 from tumor cells, indirectly increasing the infiltration of Tregs [153]. Blocking CAF-S1 using an anti-CD73 antibody reduces immunosuppression by preventing the expression of immune checkpoints (PD-1 + and CTLA-4 +) in Tregs [154]. In addition, apCAFs, stimulated by IL-1 and TGF-β signaling, represent a unique immunoregulating CAF population that can induce Treg formation and expansion through antigen-dependent TCR ligation in pancreatic cancer [34]. Of note, CAF-derived IL-6 can induce CD73( +)γδ Tregs to differentiate and secrete more adenosine, thereby establishing a positive feedback loop that greatly weakens the anti-tumor effects of CD8 + T cells and leads to a worse prognosis in patients [155]. Surprising, however, Özdemir et al. [6] reported the exhaustion of myofibroblasts in PDAC increases the proliferation of CD4 + Foxp3 + Tregs, subsequently inhibiting immune surveillance. Overall, CAFs and Tregs appear to have a complicated and dual connection, potentially influenced by the specificity of cancer and heterogeneity of CAFs. Therefore, further research is needed to explore the relationship between CAFs and Tregs across different malignancies.

It is well established that CD8 + T cells are essential for anti-tumor immunity, and the efficiency of immunotherapies depends on the considerable infiltration of CD8 + T cells into the tumor [156]. Various studies have demonstrated the inhibitory effects of CAFs on CD8 + T cell infiltration [83, 157–160], proliferation [161], anti-tumor immunity [162–164], and promotion of apoptosis [138]. The immunosuppressive effects of FAP + CAFs in multiple malignancies have also been described, with their depletion enabling effective tumor progression control [48–50, 165–168]. Furthermore, CAFs can also limit CD8 + T cell recruitment and infiltration by producing IL-6 [169, 170], TGF-β [171], and CXCL12 [168, 172], and inhibiting their cytotoxic capabilities against tumor cells. Goehrig et al. [163] found that CAFs can directly suppress CD8 + T cell proliferation, activation, and cytotoxic activity through the interaction of CAF-secreted βig-h3 (also known as TGF-βi) with CD61, a CD8 + T cell surface marker, resulting in reduced TCR signaling transduction. Physical barriers and hypoxia in the TME caused by CAF-mediated ECM alterations can also limit T cell migration [157, 173]. Inhibition of the Hedgehog pathway can lead to a decline in myCAFs and an increase in iCAFs, accompanied by a reduction in cytotoxic T cells and an elevation in Tregs, indicating heightened immunosuppression [174].

#### Regulatory effects of CAFs on other immune cells

Other immune cells, such as B cells, may also be affected by CAFs. To date, however, only the effects of CAF-secreted CXCL13 on B cell recruitment have been reported [175], with limited research on other interactions between CAFs and B cells.

In summary, reciprocal interactions between CAFs and other immune cells contribute to the modulation of phenotype and function, shaping the diverse outcomes in tumor occurrence and progression. However, there is still a large gap in our understanding of the interconversion between CAFs and other cells within the TME, which presents a promising novel direction for future research.

### Tumor-suppressive functions of CAFs

Various studies have provided evidence emphasizing the context-dependent tumor-suppressive functions of CAFs. The mechanisms by which CAFs exert tumor-suppressive functions involve the promotion of anticancer immunity, activation of tumor-suppressive signaling, and production of ECM components to hinder tumor cell invasion and dissemination.

Deletion of αSMA + CAFs in a mouse model of pancreatic cancer has been shown to accelerate tumor growth, reduce fibrotic response, and lower survival [6]. Targeting the Sonic hedgehog (SHH)-Smoothened (SMO) signaling axis has also been shown to increase cancer cell proliferation and tumor formation by inhibiting SHH-SMO-mediated activation of the tumor-suppressive phenotype in myofibroblasts [104, 176]. These findings suggest that the tumor-suppressive functions of αSMA + CAFs are mediated, at least in part, via the SHH-SMO signaling pathway.

In preclinical models of pancreatic cancer, JAK inhibitors have been found to inhibit LIF signaling in IL-1-induced inflammatory iCAFs, thereby converting iCAFs to ECM-producing myCAFs, increasing the myCAF-to-iCAF ratio, and promoting ECM deposition, leading to a reduction in cancer cell proliferation and tumor growth [8]. In addition, specific subgroups of CAFs with specific markers have been shown to have tumor-suppressive functions, although the precise mechanisms underlying their anti-tumor effects is unclear [44].

The discovery of the tumor-suppressive functions of CAFs provides a potential explanation for the failure of clinical trials targeting CAFs and stromal components. These observations suggest that future therapeutic strategies should avoid generalized targeting of protumorgenic CAF subpopulations in favor of precise reprogramming and promotion of normalization or conversion of tumor-promoting CAF subsets to tumor-suppressive CAF subsets.

## Strategies targeting CAFs

**Fig. 4:**
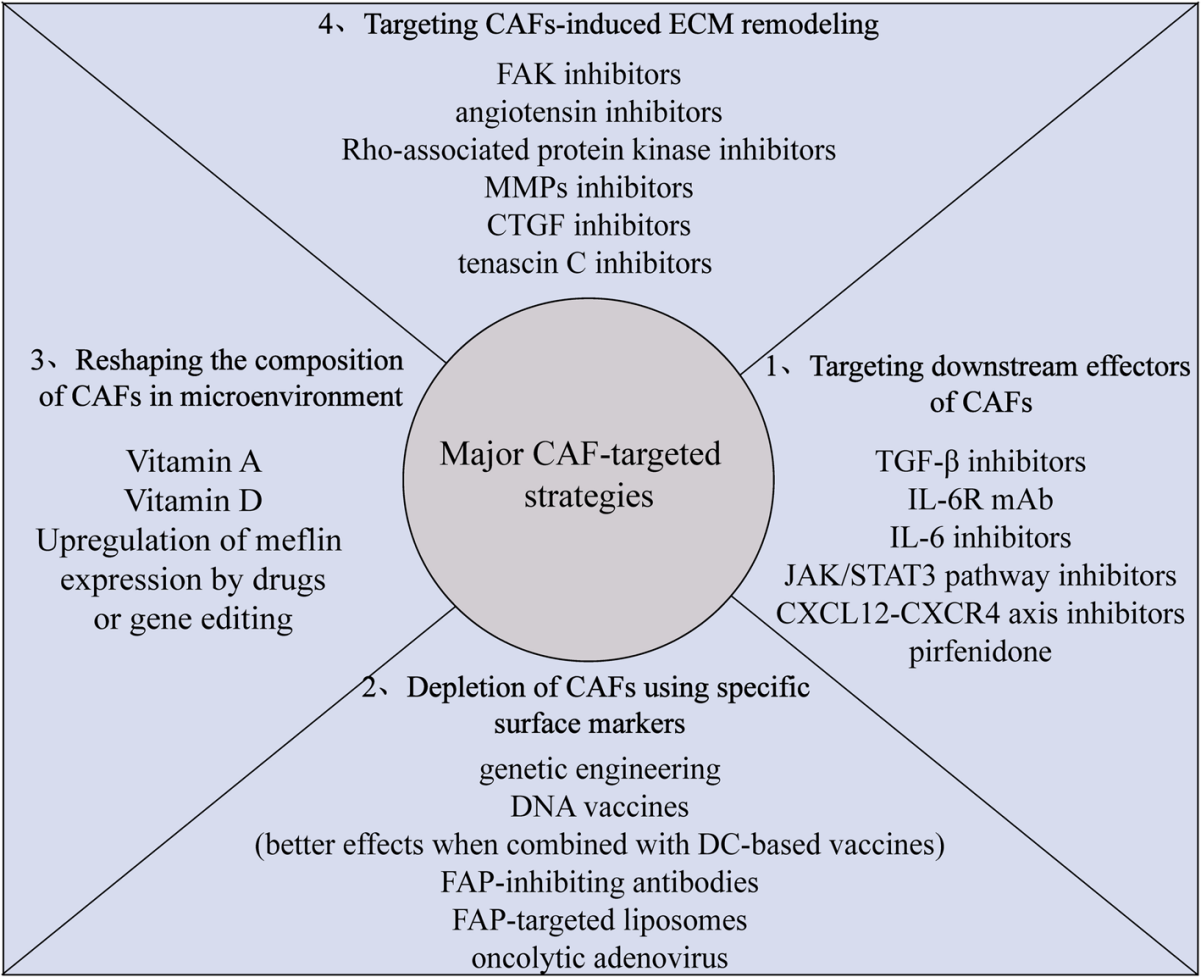
Principal strategies for targeting CAFs. The primary anticancer agents that target the stromal compartment in malignancies are printed. The activation or functions of CAFs can be inhibited by targeting important signals and effectors of CAFs, such as chemokine and growth factor pathways. Moreover, either transgenic technologies or immunotherapies can directly deplete CAFs. Through the application of chemicals like vitamin A or vitamin D, CAFs can also be adjusted to an inactive phenotype. Finally, it is viable to target CAFs-derived extracellular matrix proteins and related signaling to deplete the stroma and boost immunological T cell infiltration

CAFs are implicated in tumor occurrence and growth, primarily through their role in creating a suppressive TME, making them a promising target for tumor treatment. However, CAF-targeted therapies face considerable challenges, including the lack of specific surface markers, which may lead to various non-specific side-effects or even promote the development of malignant tumors. Comprehensive characterization and functional validation of CAF subtypes may pave the way for the development of innovative CAF-based diagnostic and therapeutic approaches. Here, we provide a brief discussion of existing therapies that specifically target CAFs to explore potential synergistic combinations that may enhance clinical prognosis for patients (Fig. 4).

### Targeting downstream effectors of CAFs

CAFs play a crucial role in creating an immunosuppressive TME by secreting various cytokines and chemokines that trigger specific downstream signaling pathways. Consequently, novel therapeutic approaches have been proposed to target downstream effectors of CAFs, with the aim of promoting immune cell infiltration and enhancing anti-tumor capabilities. For example, combination treatments involving TGF-β inhibitors and anti-PD-L1 immunotherapy can reduce TGF-β signaling in stromal cells, leading to increased T cell infiltration, and improved anti-tumor immunity [177]. Elevated levels of IL-6, which is secreted by activated CAFs, particularly FAP + CAFs, contribute to the proliferation of immunosuppressive cells, impairment of anti-tumor cell functions, and ultimately, tumor progression and therapy resistance through various downstream signaling pathways [17, 49, 118, 158, 173, 178]. Thus, agents targeting the IL-6, IL-6R, or JAK/STAT3 signaling pathways show promise as potential candidates for combined therapy [178–180]. The CXCR4 antagonist motixafortide (BL-8040), which can inhibit the immunosuppressive CXCL12-CXCR4 axis driven by FAP + CAFs, is currently being investigated in phase II clinical trials involving pancreatic cancer patients in combination with pembrolizumab and/or chemotherapy (NCT02826486). Pirfenidone treatment has been shown to reduce PD-L1 expression and CCL17 and TNF-β secretion in CAFs, thereby reducing the acquisition of CAF-mediated invasive and immunosuppressive functions in breast carcinoma cells [181]. In general, drugs that target stromal CAF signals and effectors have emerged as important additions to anti-tumor therapy, especially in combination with immune checkpoint inhibitors.

### Depletion of CAFs using specific surface markers

CAF-targeted therapies have primarily focused on CAF depletion by targeting specific surface markers. Notably, FAP has emerged as a practical target molecule for clinical applications [182]. Genetic depletion of FAP allows considerable immunological control of tumors due to the rapid hypoxic necrosis of both cancer and stromal cells via IFN-γ and TNF-α, leading to enhanced infiltration and anti-tumor capacities of T cells [168, 183]. Moreover, FAP-based DNA vaccines have been developed as a principal type of CAF-targeted therapy [184]. Combining anti-CAF therapies and DC-based vaccines can elicit broad T cell responses and potent anti-tumor activity, as well as a decrease in the infiltration of immunosuppressive cells [185]. FAP-targeted techniques, such as FAP-CAR-T cell therapy [186], FAP-targeted oncolytic adenovirus [187], FAP-inhibiting antibodies [188], and FAP-targeted liposomes [189], can also promote specific immune attacks against FAP + CAFs, which is effective against tumors.α-SMA is another critical marker of CAFs [190]. Depletion of α-SMA + CAFs can impede cancer cell dissemination and tumor angiogenesis in BC and PDAC models [6, 191] and is associated with increased disease aggression and progression by enhancing CD3 + Foxp3 + Treg infiltration in the TME [6]. Depletion of α-SMA + fibroblasts is also reported to lead to more aggressive tumors and shorter overall survival, indicating that these cells may play a role in preventing pancreatic cancer progression [192]. However, further research is needed to determine their dual effects on tumor progression and better design CAF-targeted therapies.

In mouse models, the elimination of CD10 + GPR77 + CAFs, a specific CAF subgroup linked to chemoresistance and poor survival in breast and lung cancer, can result in decreased tumor development and increased chemotherapy effectiveness [48]. In addition, a mAb-targeting mesothelin effectively inhibits apCAF formation and reduces the Treg/CD8 + T cell ratio [34]. However, more highly selective markers are required to improve the accuracy of CAF-based therapies.

### Reshaping CAF composition in the microenvironment

In addition to direct depletion of pro-tumorigenic CAFs, new CAF-targeted strategies have been developed to convert their active state into a quiescent state or a tumor-suppressive phenotype, and thus establish a more favorable environment for tumor eradication. For instance, retinol replenishment can restore quiescence in pancreatic stellate cells and increase apoptosis in neighboring cancer cells [193]. Similarly, vitamin D treatment normalizes the activated phenotype of stromal cells [194], while Minnelide actively depletes reactive stromal fibroblasts and triggers tumor regression and increased drug delivery [195]. Since the identification of CAF subclasses with the capacity to limit tumor growth [43], researchers have attempted to utilize this trait in clinical applications. Up-regulating the expression of the glycosylphosphatidylinositol-anchored protein Meflin, a rCAF-specific marker, by genetic and pharmacological approaches can improve chemosensitivity in a mouse model of PDAC [196]. Furthermore, recently discovered primary human lung apCAFs from ATII can directly prime TCRs for tumor infiltrating CD4 + T cells, which not only enhances anti-tumor immunity but also protects against apoptosis [197]. Whether stimulatory and non-stimulatory apCAFs exist within the same tumor, as well as their potential interconversion and influencing factors on function, may represent a new therapeutic direction. Understanding how CAFs are regulated by other variables in the TME during tumor development would also greatly aid in the discovery of therapeutic approaches aimed at modifying CAF composition.

### Targeting CAF-induced ECM remodeling

CAF-induced ECM remodeling serves as a physical barrier that hinders anti-tumor immune cell access and therapeutic drug delivery. Several therapeutic approaches have been designed to target CAF-derived ECM modifications. For instance, the specific FAK inhibitor VS-4718 has been shown to increase immune surveillance by overcoming the fibrotic and immunosuppressive TME, thereby sensitizing tumors to immunotherapy [198]. The angiotensin inhibitor losartan reduces stromal collagen content and hyaluronan production, thereby potentiating chemotherapy in breast and pancreatic cancer models [199]. Similarly, the Rho-associated protein kinase inhibitor, which targets the ECM [200], and other strategies targeting connective tissue growth factor (CTGF) [201], tenascin C [202], and matrix metalloproteinases (MMPs) [203], may improve clinical effects. In summary, modification of the ECM after treatment can alleviate the suppression of immune effector cell recruitment into tumors, thus enhancing anticancer immunity and improving therapy resistance.

## Discussion and further expectations

CAF-based research has entered an exciting and crucial phase, revealing their complex and diverse functions. Regarding CAFs as uniformly tumor-promoting or tumor-suppressing populations greatly underestimates their complexity. The heterogeneity of distinct CAF subgroups at the single-cell level will be increasingly explored using cutting-edge technologies such as scRNA-seq and single-cell analysis. These approaches will also play a crucial role in precisely classifying and characterizing various CAF subgroups at the single-cell level, providing valuable insights into their diverse cellular states and functions. However, the current lack of a unified and standardized system for classifying CAFs remains a challenge. Indeed, the subjective nature of defining CAF subpopulations has led to varied definitions, even within the same malignancy. Thus, researchers will need to establish a consensus on the major biomarkers and hierarchical clustering of CAF subclasses based on scRNA-seq technology. This will facilitate better identification of CAF subclasses with specific functional and prognostic values, leading to targeted treatment approaches for precise CAF subclasses and a better understanding of their associated molecular mechanisms in clinical settings.

Despite significant advancements in our understanding of CAF biology, several important questions remain unresolved. The identification of various CAF subtypes raises the question of whether they represent distinct lineages or different functional states of the same cell population, capable of interconversion depending on their surrounding microenvironment niche. For instance, the abundance of PDPN + CAFs decreases while that of S1004A + CAFs increases with BC progression [4], and these two CAF subtypes can be further split into many functional subclasses. Although changes in the composition of CAF subclasses during tumor evolution have been observed in cancer research, the underlying mechanisms driving these changes are not yet clear. Furthermore, numerous questions remain to be answered. For example, how are CAFs regulated within the TME? What are the specific molecular mechanisms that govern the differentiation of CAFs into functional subtypes? How do these functionally distinct CAF subgroups impact the immune milieu and, consequently, anticancer immunity?

Our understanding of how different functional CAF subtypes coordinate the TME has greatly advanced, providing valuable insights for the translation of CAF-based therapeutic approaches into clinical trials. However, further investigation is required to elucidate how each CAF subtype affects the epithelial compartment and other cell types within the TME, and how different subtypes coordinate with each other to control tumor progression.

## Conclusions

This review provides an overview of the multilevel heterogeneity of CAF molecules and functions, emphasizing their dynamic regulation and morphological and functional changes within the TME. Future research using new technologies to describe the unknown aspects of tumors in more precise detail and to explore the interactions and mechanisms between CAFs and other types of cells in the TME will yield great benefits. These efforts will lay the foundation for accurate oncology and improved patient prognosis by identifying relevant CAF targets. Finally, this review highlights the significance of targeted CAF therapies and their potential combination with immune checkpoint blockade to enhance therapeutic responses.

## Data Availability

Not applicable.
